# Impact of Nighttime Food Consumption and Feasibility of Fasting during Night Work: A Narrative Review

**DOI:** 10.3390/nu15112570

**Published:** 2023-05-31

**Authors:** Luisa Pereira Marot, Tássia do Vale Cardoso Lopes, Laura Cristina Tibiletti Balieiro, Cibele Aparecida Crispim, Cláudia Roberta Castro Moreno

**Affiliations:** 1 Chrononutrition Research Group, Faculty of Medicine, Federal University of Uberlândia, Uberlândia 38405-320, Brazil; luisamarot@hotmail.com (L.P.M.); tassia_dovale@hotmail.com (T.d.V.C.L.); laura.balieiro@hotmail.com (L.C.T.B.); cibelecrispim@gmail.com (C.A.C.); 2 Department of Health, Life Cycles and Society, School of Public Health, University of São Paulo, Sao Paulo 01246-904, Brazil

**Keywords:** shiftwork, eating habits, mealtime, fasting, chrononutrition

## Abstract

Shift work has been associated with an increased risk of developing chronic non-communicable diseases, such as obesity. The reduction in overnight fasting and its physiological consequences seem to affect the metabolic health of shift workers, but little has been discussed regarding the feasibility and implications of maintaining a night-long fast during work. This narrative review aims to discuss the impact of eating behavior on the reduction of overnight fasting in shift workers, as well as possible nutritional strategies involving fasting that have been tested for shift workers, to contribute to the establishment of nutritional guidelines for them. We used various databases and search engines to retrieve relevant articles, reviews, and investigations. Despite the potential benefits of overnight fasting for other groups, few studies have investigated this approach in the context of shift work. Generally, it seems to be a feasible and metabolically beneficial strategy for shift workers. However, it is essential to investigate the potential risks and benefits of reducing the fasting time for shift workers, considering social, hedonic, and stress-related factors. Furthermore, randomized clinical trials are necessary to establish safe and feasible strategies for shift workers to practice different fasting windows.

## 1. Introduction

Shift work is increasingly common and necessary in modern 24/7 society. The prevalence of this type of work schedule varies between 15–20% of the workforce around the world [1,2]. Several studies have demonstrated that shift workers have a higher risk of developing chronic non-communicable diseases such as obesity [3,4], metabolic syndrome [5], and cardiovascular diseases [6]. Among the different causes of the relationship between shift work and these diseases are circadian misalignment [7], sleep restriction [5,8,9] and lifestyle habits that deteriorate with exposure to work at atypical hours [10,11,12]. How much each of these problems contributes to metabolic diseases and the potential way in which they interact is unclear so far.

Importantly, the temporal misalignment between light–dark and wake–sleep, eating–fasting and activity–rest cycles [7] has been associated with metabolic dysregulation that controls food intake [13,14,15,16]. Thus, food consumption is among the modified life habits of shift workers [17,18,19], which is also influenced by the social harm resulting from shift work. Studies on this topic have shown that shift workers, especially night shift workers, tend to have a poor diet composed of meals of low nutritional quality [20,21,22] compared to day workers, which are often consumed late at night [10,11,12]. In this way, the extended periods of wakefulness during night shifts seem to contribute to an extended eating window and, consequently, to higher energy intake [11]. Studies have shown that shift workers consume a significant part of their meals at night [23,24], in a period of poor postprandial metabolism [25,26]. Consequently, the physiological overnight fasting is substantially reduced [24].

From a physiological perspective, the fasting period is essential, since this is when occurs shifts on the metabolic processes, cellular repair and restoration of redox states [27]. Faced with changes in the food consumption of shift workers, it is reasonable to assume that the lack of overnight fasting impairs the metabolism function and nutritional status of these workers. On the other side, fasting all night at work seems to be a challenging strategy for shift workers, considering that many of them eat for hedonic reasons, for socialization or even to stay alert [28]. Unfortunately, the practice of fasting with the aim of improving health—which has recently gained notoriety with interventions such as intermittent fasting and time restricted eating [29,30,31,32,33,34]—has not often been addressed in studies with shift workers. In general, there are substantial questions about “what to eat” and “when to eat” for shift workers, and both points seem to impact shift workers’ fasting time.

This narrative review aims to discuss the impact of the eating behavior on the reduction of a night fasting period in shift workers, as well as possible nutritional strategies involving fasting that have been tested for shift workers in order to contribute for establishing nutritional guidelines for shift workers.

## 2. Methods

A comprehensive literature search was conducted to identify relevant publications on the shortening of the night fasting period in shift workers, as well as nutritional interventions involving fasting for these individuals. Different databases (e.g., PubMed) and search engines (e.g., GOOGLE SCHOLAR) were utilized to retrieve articles, reviews, and investigations. The search used Boolean operators AND/OR, with the following keywords: shift work, fasting, mealtime, night intake, chrononutrition.

Inclusion criteria encompassed original articles published in English and conducted in humans, either in simulated shift work or under real-life conditions. Excluded from the selection were case reports, case series, letters to editors, commentaries, editorials, critical reviews, systematic reviews and/or meta-analyses, and articles lacking new objective data. The references cited in critical reviews, systematic reviews, and/or meta-analyses were screened for additional eligible articles. No temporal limits were set, and the search considered articles published up to April 2022. Articles that did not align with the review aim and predetermined inclusion criteria were rejected.

The terms used identified 324 hits. Duplications were removed, and a further evaluation of the relevance of each article was performed. Three investigators (LPM, TVCL, LCTB) independently screened potentially eligible studies by evaluating titles and abstracts for agreement with the inclusion criteria. Subsequently, the same investigators independently read the full articles considered potentially relevant in their entirety. Any disagreements were solved through discussion among the investigators. At the end of this process, a total of 14 articles were identified and included in the present review.

## 3. Physiological and Chronobiological Bases That Regulate the Eating–Fasting Behaviors in Humans

The circadian timing system is a complex system made up of an endogenous clock network, involving a central clock and peripheral clocks. The central clock is the suprachiasmatic hypothalamic nucleus (SCN), regulated by environmental light signals, and which acts as a pacemaker for the production and maintenance of circadian rhythm throughout the body [35]. Identification of light by the retina and transduction to the SCN allows the synchronization of tissue activities and behaviors with day/night cycles through the clock genes present in cells in tissues [35]. Clock genes regulate the transcription of clock-controlled genes, which control the timing of tissue-specific functions [36,37]. Studies in vitro and with mice have pointed out local tissue-specific processes controlled by peripheral clocks in several organs, such as the regulation of fasting glycemic control and glucose clearance by the hepatic clock [37], regulation of insulin secretion and its response to glucose by the pancreatic clock [38], regulation of lipid storage and mobilization by the adipose clock [39], and regulation of glucose uptake and metabolism by the skeletal muscle clock [40]. Once peripheral clocks have not only synchronized to the SCN [35] but also to other exogenous components—such as food intake [41,42]—the metabolic feedback related to these clocks can be mediated through circadian–endocrine crosstalk [43].

The activity phase of the day for humans generally begins around sunrise and ends at bedtime, when the rest phase that occurs during the dark phase of the day begins. Importantly, a strict physiological control works to adjust the circadian timing system to external environmental cues providing energy for periods of activity and saving energy during the rest period [12]. From this perspective, overnight fasting is not only expected by the organism, but represents a necessary break that synchronizes our peripheral circadian timing system.

The circadian timing system controls energy metabolism, once several genes related to glucose and lipid homeostasis are under circadian control [44]. In the postabsorptive state, the reduction in the energy availability increases 5AMP-activated protein kinase (AMPK) phosphorylation in order to raise ATP formation; AMPK interacts with the NAD-dependent protein deacetylase SIRTUIN (SIRT) 1, which together with SIRT6 controls the rhythmic genes transcription in the liver, with genes involved in peptide and cofactor metabolism regulated by SIRT1 and genes related to carbohydrate and lipid metabolism regulated by SIRT6 [45]. In this way, metabolic hormones, circulating nutrients, and visceral neural inputs transmit rhythmic cues that synchronize brain and peripheral organs to eating time [12]. Among the several hormones that participate in the circadian control that determine that food intake and fasting occurring during the day and night, respectively, is melatonin, whose oscillations have a particular characteristic pattern commonly used to define daily cycles [46]. Melatonin secretion depends on the photoperiod and can be described as a chemical expression of darkness, as it is produced during the night in response to less exposure to light [47,48]. This hormone is responsible, in part, for the daily distribution of metabolic processes so that the activity/eating phase of the day is associated with high insulin sensitivity, and rest/fasting is synchronized to the insulin-resistant metabolic phase of the day [49]. In contrast to melatonin is another hormone, cortisol. At the beginning of the day’s activity, the body is prepared to wake up due to a cortisol peak, a hormone that prepares the body for the increase in energetic demands induced by activity [50], and whose production is associated with decreased melatonin secretion [51]. Some hormones act directly to food intake such as ghrelin and leptin. Ghrelin is an important hormone that regulates energy homeostasis by increasing appetite and food intake. Secreted primarily by cells in the stomach, ghrelin levels follow a pulsatile rhythm around 8:00 h, 13:00 h, and 18:00 h [52,53], stimulating food intake in these times. In the same way of ghrelin, leptin also has a pulsatile pattern, but it shows a nocturnal rise [54]. This characteristic of leptin is compatible with the stimulation of energy homeostasis, suppression of food intake, increased lipolysis and inhibition of fat accumulation [50]. Another important regulator of nutrient metabolism is adiponectin, which begins secretion at 10:00 h and ends at 20:00 h, with a peak secretion level attained at 11:00 h [50]. This hormone improves glycolysis and fatty acid oxidation via the activation of AMPK, a kinase involved in the support of energetic homeostasis, and which also reduces hepatic glucose production. These mechanisms increase glucose use and insulin sensitivity besides preventing fat accumulation [55]. Finally, it is essential to emphasize diurnal oscillations in insulin and nutrient metabolism and their impact on the metabolic health of human beings. Insulin activity shows significant variations throughout the day, with marked impairment at night [56]. These diurnal variations in glucose tolerance can be partially traced to diurnal rhythms in β-cell responsiveness, insulin secretion, and insulin clearance. In general, β-cell responsiveness—as measured by glucose tolerance or, mixed meal, or intravenous tolbutamide testing—is higher in the morning than at other times of day [57,58,59,60,61]. For example, insulin levels are reduced to the offset in the resting phase, which results in a lower beta-cell responsivity to glucose [26]. In this regard, the body will not be able to process glucose properly during the evening, leading to lower insulin sensitivity [56,62]. Recently, it has been demonstrated that some intestinal functions are rhythmically regulated. Signals from intestine provide information regarding food availability to dorsomedial hypothalamus, which might influence other tissue and regulate food anticipation, digestion, and absorption [26].

Based on the above, it is possible to observe that the human being has a hormonal and physiological daytime pattern compatible with hunger, appetite, food intake and metabolism during the day and fasting at night. Emerging evidence on the intersection between mealtime and energy metabolism suggests that mealtime may influence metabolism and have implications for body weight regulation and energy metabolism [63,64]. For example, irregular eating patterns, such as skipping meals or consuming a large number of calories late at night, have been linked to metabolic dysregulations, including an increased risk of obesity, insulin resistance and metabolic dysfunction [63,65]. From this perspective, the alignment of food intake schedules with the body’s circadian rhythm has gained prominence in the field of chrononutrition [64]. Studies in this area indicate that nutritional strategies such as time-restricted eating—which adopts a limited eating window during the day—may have beneficial effects on body weight regulation, glucose and lipid metabolism, and general metabolic health [42,66]. In this regard, potential mechanisms involved in the crosstalk between mealtime and energy metabolism include circadian timing regulation, hormone secretion, and metabolism-related gene expression modulation [67,68].

In this entire scenario, when the shift worker consumes meals outside the hours expected by the body, there is a conflict between the endogenous rhythm of nutrient metabolism and the external cue [69]. Such a conflict between metabolic functioning, food intake and fasting seems to be an important pathway capable of leading to metabolic damage and obesity. A study by Mchill et al. (2017) [70] with non-shift workers showed that higher percentages of body fat were correlated with the percentage of calories consumed between 4 h before DLMO until bedtime, and negatively associated with the caloric midpoint time in relation to DLMO. It is also already known that the extended waking periods to which shift workers are exposed facilitate this eating pattern of night meals [11,12] and skipping breakfast [28], a habit associated with negative effects to health, such as overweight/obesity [71].

Taken together, the above information leads us to believe that the eating schedules of shift workers should be aligned with our functioning predicted by our circadian physiology (i.e., eating during the day and fasting during the night) [12,72]. However, there are numerous challenges of restricting the food intake of workers during the night, which will be addressed throughout this article.

## 4. What Is the Main Problem of Shift Workers Eating Habits: “What” or “When” Do They Eat?

Excessive energy intake [10], poor food quality [20,21,73], and eating late at night [12,24] are problems related to the dietary intake of shift workers commonly reported in the literature. From a nutritional point of view, so far it has not been possible to establish whether the high prevalence of overweight/obesity in these individuals is due to energy and poor intake, often excessive, which is the result of the greater opportunity to eat because they are awake for more time; the decrease in the fasting period, which can lead to lower lipolysis and lipid oxidation [26]; nocturnal metabolic impairment associated with food intake; or all of these factors interacting.

The eating habits of shift workers have been increasingly studied in recent years [10,12,24,28,74,75]. From a quantitative point of view, it seems that energy intake among shift workers does not differ from day workers [75], nor even when comparing the day shift, night shift and days off of rotating workers [10,12,24]. However, studies evaluating real-life shift workers with the aim of estimating energy intake have been performed with heterogeneous samples from various work activities and different work schedules [28,75]. Furthermore, in general, these studies have not used the same method for dietary assessment and the sample size has often been reduced [28]. Thus, it is not possible to draw definitive conclusions about the amount of food consumed by these individuals. Furthermore, the possibility of underreporting of dietary intake should always be considered in general populations, especially those vulnerable to obesity [76], such as shift workers. Therefore, it is evident that this is an area of research that needs further study.

Although a systematic review with meta-analysis showed that there was no difference in 24 h energy intake between night and day workers [75], previous studies have shown that the quality of food consumed by shift workers seems to be worse than day workers, with a low frequency of sources of fiber, such as fruit and vegetables, important components of a balanced diet, and high consumption of food rich in fat and sugar, caloric foods [20,21,22]. In a cross-sectional study of bus drivers, Balieiro et al. (2014) [20] showed that night workers presented a higher proportion of inadequate consumption of vegetables (lower) and fat (higher) when compared to day workers. Hemio et al. (2015) [22] evaluated three working groups from an airline company: day work, shift work without in-flight work and in-flight work, and showed that among women, the proportion of energy derived from saturated fat was higher for shift workers compared to day workers. Fernandes et al. (2013) [21] found similar results when evaluated 2279 nurses from 18 Brazilian public hospitals; they demonstrated that the longer workdays favor greater chances of consuming fried foods. By evaluating the components for Adjusted Healthy Eating Index (AHEI), Mota et al. (2013) [77] showed that resident physicians present low intake of vegetables (4.8 ± 2.8) and fruits (4.8 ± 3.8) and the high intake of sweets (5.3 ± 4.1), saturated fat (5.7 ± 3.8) and cholesterol (8.8 ± 2.4) [63]. Evaluating several shift schedules, Bucher Della Torre et al. (2020) [78] found that the consumption of fruits and vegetables were lower on the recovery day (day following night shift) (1.99 ± 1.42) than day shifts (2.85 ± 2.07; *p* = 0.001). On the other hand, unlike in previous studies, Shaw et al. (2019) [12] evaluated night shift workers with several different occupations at different days of the shift schedule and did not find differences in the consumption of fruits, vegetables, whole grains, dairy products, meat, caffeine, and alcohol, comparing the different days of the shift.

Against the backdrop of energy intake equivalence in 24 h and some qualitative differences, some studies have recently begun to investigate the temporal distribution of meal consumption [10,12,23,24], the eating window [10,11,23] and its association with energy intake. According to a review conducted by Gupta et al. (2019) [28], while day workers present a pattern of 3 meals in a 24 h period, shift workers have difficulty maintaining a regular pattern of meals and times. Night shifts, regardless of whether they are fixed or rotating, favor longer eating windows and, consequently, more opportunities to eat over a 24 h period, since shift workers fit their eating and sleeping schedules according to work schedules [10,11,12]. Across shifts, the eating window is aligned with the waking time; the longer the waking time, the longer the eating window and the greater the calorie consumption [11]. The amount of energy intake seems to be associated with the eating window, as we showed in previous studies conducted from our research group [11,23]. Studying rotating shift workers from a miner company, we found that the wakefulness promoted by the transition from afternoon to night shifts leads to the largest eating window of the schedule (20.7 ± 1.2 h) and, consequently, the highest energy intake (3410 ± 235 kcal) at the same period [11]. In another study, we evaluated time-related eating patterns of day and night military police officers and showed that those shift workers with later caloric midpoint, both day and night shift workers, had higher energy intake compared to earlier eaters. However, when compared with day workers, night workers had a later caloric midpoint, which is a consequence of the extended period of wakefulness promoted by the night shift [23].

Shaw et al. (2019) [12] evaluated workers from different areas (transport, health and others) and demonstrated that, when working night shifts, workers tend to eat ‘24 h a day’ without a fasting period. In the same line, Kosmadopoulos et al. (2020) [10] demonstrated that on night shift days of US police officers, meals are significantly more dispersed over a 24 h period than on all other days of the rotating shift schedule. A study with Brazilian police officers showed that night workers usually had their last meal later, between 23:00 h and 4:59 h, while day workers fished their eating day between 17:00 h and 22:59 h [23]. In addition, clockwise rotating shift workers had 17% and 14% of their total calories of the day between 00:00 h and 3:59 h when working in the first and second days of night shift, respectively, while the most of the other shift days of the schedule remained less than 3% of the total calories in the same time range [24]. However, it seems that workers tend to return to more typical eating patterns (breakfast, lunch and dinner) and 8 h of overnight fasting on days off [12,24], presenting a food window more aligned with the light period of the light–dark cycle [11]. Thus, longer eating windows favor night meals [10,12]. From a health perspective, it is already known that non-shift workers who prioritize energy intake later in the 24 h day are more likely to be overweight [79]. On this topic, McHill et al. (2017) [70] found that obese individuals consumed most of their calories an hour closer to melatonin onset—strongly regulated by light/dark cycle with high levels during the night—compared to lean individuals.

Given the above, it is presumed that the high prevalence of metabolic diseases in shift workers may, at least in part, be associated with the fact that food intake occurs frequently and often in high volume and poor quality, at a time where the body is not programmed to metabolize nutrients [80,81]. However, there is a clear need for additional randomized controlled trials to test the effect of overnight eating/fasting on nutritional and metabolic outcomes.

## 5. Fasting to Prevent and Treat Nutritional Diseases

Fasting is defined as the removal of solid foods as well as carbohydrate, protein, or fat-containing beverages over a specific timespan. The period of physiological fasting, which primarily occurs during the nocturnal phase of the day, typically lasts between 8 and 14 h [82]. From a physiological perspective, the period of fasting is essential, as changes in metabolic processes, cell repair and restoration of redox states occur during this period [27]. Fasting affects substrate metabolism, stimulating fat metabolism and the metabolic switch between glucose oxidation and fat oxidation [26]. In this sense, during nocturnal fasting there is an increase in TG (triacylglycerol) lipolysis highlighted by the increase in plasma FFA and glycerol [83]. It follows that fat metabolism will be more active during the night and fat oxidation takes place mainly at this time [84,85]. Thus, with the diurnal nature of humans to be awake during the day and sleep at night, it is expected that daytime food intake would be associated with food intake followed by glucose metabolism and fat deposition, and nocturnal fasting with fat metabolism. This balance between storing and expending fat over a 24 h period gives physiological overnight fasting an important role in controlling body weight in humans. However, the real impact of lack of fasting due to shift work has been little addressed.

Many current clinical research protocols in non-shift workers have shown that extending the fasting period has physiological and metabolic benefits [29,30,32,33,86,87]. A commonly tested protocol in this regard and time-restricted eating (TRE), which encompasses recurrent fasting periods on a daily basis, ranging from 12 to 21 h, followed by ad libitum food and fluid intake for the remaining hours of the day [34]. Studies have shown that early time-restricted eating, where the food intake occurs between 06:30 h and 18:00 h, facilitates weight loss and appetite reduction in overweight and obese people [29,30,32,33]. In addition, prediabetic and diabetic patients presented lower insulin levels and a better insulin sensitivity when they restrained their daily eating window to 8 h per day [30,33,88]. Moreover, it induced changes in cardiovascular markers by decreasing blood pressure and LDL levels [29,33]. Chew et al. (2022) [86] conducted an umbrella review aiming to summarize systematic reviews that examine the effects of TRE on weight loss, fasting blood glucose, total cholesterol, triglycerides, high-density-lipoprotein cholesterol (HDL-C) and low-density-lipoprotein cholesterol (LDL-C) in individuals with overweight and obesity. Seven systematic reviews with 30 unique meta-analyses involving 7231 participants from 184 primary studies were included. Meta-analysis at the meta-data level suggested that TRE was beneficial for reducing weight and fasting blood glucose, and may complement usual care and reduce body weight and fasting blood glucose. Unfortunately, shift workers are often excluded from lifestyle intervention studies to reduce this risk. To the best of our knowledge, there is only one study testing TRE in shift workers, which was conducted by Manoogian et al. (2022) [19] and tested the feasibility of 10 h time-restricted eating (TRE) in firefighters (results described in the next section).

Despite the many positive effects of TRE for health, it is important to draw attention to the possibility that the benefits caused by TRE occur when the aim is to align daily food intake with the circadian timing system, avoiding nighttime food intake and prolonging the rest fast period [89,90]. In general, studies have used different durations for the eating period, from 8 to 10 h, usually during the day, and recommend diverse time slots for time-restricted eating [29,32,33,91]. However, the intervention protocols tested so far seem unfeasible for shift workers and an important question arises in this regard: is it possible for night workers to spend the whole night working without eating anything?

## 6. Is Overnight Fasting Feasible for Shift Workers? Perspectives on Establishing Nutritional Guidelines for Shift Workers

Nighttime food intake has been associated with an elevated risk of long-term consequences, including metabolic disorders, insulin resistance, type 2 diabetes, and obesity [92,93]. This has been described in both epidemiological studies [94,95] and clinical trials [13,96]. Furthermore, overnight fasting represents a necessary break that synchronizes our peripheral circadian timing system and has been shown to benefit metabolic health [97]. For all these reasons, studies on eating patterns related to time have been emerging in the literature, with an important emphasis on the importance of avoiding foods late in the day 91food or fasting, which foods to choose, the ideal amounts and, mainly, meal times, generates confusion for shift workers about which behaviors to adopt.

Recent studies have shown differences in food choices among shift workers during the night shift [98,99,100,101,102,103]. A cross-sectional study of 52 drivers was conducted to evaluate dietary patterns of truck drivers, and the authors described varied eating habits among them, with three different diet patterns: (1) traditional, characterized by beans, rice, bread, coffee, tea, juices, white meat, processed meats and inverse correlation with vegetable intake, prudent and western diet; (2) prudent, characterized by root vegetables and tubers, milk and dairy, eggs, vegetable oils and olive oil; (3) western diet, which included fast foods, soft drinks, pâtés and sauces, and processed meats [98]. In another study, nurses reported that reducing the size of meals during the night shift was a strategy to minimize gastric symptoms during the night shift, and that avoiding food was a good strategy to maintain alertness [99]. Paramedics expressed concern that not eating during their shift could be harmful to their health [100]. In turn, flight attendants reported choosing to avoid eating during the night shift to improve health outcomes [101]. Overall, the consumption of different foods in small portions has been reported by shift workers [99,102,103].

What is known so far is that the reasons why workers eat it is not just about feeling hungry [28]. A recent review article on this topic described the main influences on workers’ eating behavior with regarded to eating during work were time availability, colleagues’ influence, seeking health improvements, staying alert, avoiding gastric discomfort, and stress [28]. Therefore, it is important to understand the feasibility of overnight fasting in the context of shift work.

In order to reduce the deleterious effects of nocturnal food intake by night workers, some different nutritional strategies have been proposed. A laboratory study with simulated shift work found that not eating during the night shift and redistributing meals to daytime hours can limit the metabolic consequences of shift work [104]. These results corroborate other findings that highlight that fasting or eating a small meal during the night shift can be beneficial, and that distributing nutrients during the day may help managing food intake and choices during nighttime [105,106,107]. Centofanti et al. (2017) [108] found that having a smaller snack compared to a larger snack during the night shift led to reduced glucose impairment after breakfast.

Along the same lines, a large meal compared to fasting during the night shift and consuming food during daytime hours has been shown to impair cognitive performance and mood during the night shift [97,109]. However, LeCheminant et al. (2013) [110] found no differences in total mood score or mood subscales between two experimental conditions in 29 volunteers in a crossover clinical trial which investigated the effect of overnight fasting on energy intake. On the other hand, the authors found that there was a significant, but clinically small, change in body weight (−0.4 kg ± 1.1 versus 0.6 kg ± 0.9; *p* < 0.001) in the nocturnal food restriction condition when compared to the control.

The practice of fasting for most of the night by workers in real-life conditions has been proposed by several current studies (Table 1), with two main objectives: to test whether there is any metabolic impact, and to determine what the real feasibility of this strategy is [18,19,111]. Manoogian et al. (2022) [19] conducted a randomized controlled trial with 137 firefighters who work 24 h shifts (23–59 years old, 9% female). The authors found that 12 weeks of 10 h TRE was feasible, with TRE participants decreasing their eating window (baseline, mean 14.13 h, 95% CI 13.78–14.47 h; intervention, 11.13 h, 95% CI 10.73–11.54 h, *p* = 3.29 × 10^−17^) with no adverse effects, and improved quality of life assessed via SF-36. It was also found that, compared to the standard of care (SOC) arm, TRE significantly decreased VLDL particle size. In participants with elevated cardiometabolic risks at baseline, there were significant reductions in TRE compared to SOC in glycated hemoglobin A1C and diastolic blood pressure. For individuals working a 24 h shift schedule. In a study by Leung et al. (2021) [18], night shift workers with abdominal obesity underwent 4-week intervention and control periods, separated by ≥2 weeks washout. In the intervention period, an overnight fast (01:00 h–06:00 h) was implemented by redistributing 24 h energy intake. The authors found that night shift workers who habitually ate during their night shifts were able to rearrange their meal times to maintain a small overnight fast, which may have promoted small weight changes. They also found a slight but significant weight loss [18]. Additionally, a study conducted by our group evaluated night workers under three different conditions in a randomized, crossover design: two nights of fasting during the night shift; two nights with the consumption of a standardized meal during the night shift; and two nights of sleep. We found that fasting during the night shift leads to not only a higher intake of energy and macronutrients both in the early morning after work and throughout the next day, but also lower insulin levels and HOMA-IR in the morning. These results suggested that eating a calorie-controlled meal at night could be a better nutritional strategy in terms of promoting energy deficit and, therefore, more viable to treat the nutritional problems frequent in these workers, such as obesity [111].

Despite the works mentioned above, it is important to recognize the current gaps in the literature on the subject and the need for further studies in order to understand whether the worker should really eat or fast during the night and, if they have to eat something, how this meal should be and the possible consequences of this nocturnal consumption on food intake and metabolic parameters on the following day. Gupta et al. (2019) [105] suggested that a small snack representing just 10% of the recommended daily energy requirement may be a more appropriate and realistic option for night and shift workers. According to the authors, a meal with limited energy content may be necessary for individuals who work all night in some work modalities to provide energy to work and to contribute to the hedonic sensations related to food, which comprise the functions of food intake in its broadest sense. However, it remains unclear how food intake should be advised for night workers, whether or not night workers should eat at night, and the effects of night food intake on food intake the next day.

## 7. Perspectives and Conclusions

The reduced physiological fasting of night shift workers appears to be inadequate for the circadian metabolic functioning of human beings. Despite the potential benefits of overnight fasting, few studies have investigated this approach in the context of shift work. Thus, it is essential to investigate the potential risks and benefits of reducing the fasting time for shift workers, taking into account social, hedonic, and stress-related factors. Additionally, it is important to conduct epidemiological studies to better understand the eating habits and fasting times of different classes of shift workers and their relationship with nutritional diseases. Moreover, randomized clinical trials are necessary to establish safe and feasible strategies for shift workers to practice different fasting windows. Given the high prevalence of shift work in our society and the well-documented link between this type of work and metabolic diseases, it is crucial for the nutrition scientific community to develop specific nutritional guidelines for shift workers. These concepts should be incorporated into the work of nutrition and occupational health professionals who deal with shift workers, to promote metabolic health and overall well-being.

## Figures and Tables

**Table 1:** Studies investigating overnight fasting or TRE as a strategy for shift workers under real life conditions.

Authors, Year	Participants Information	Shift-Type	Intervention	Results
Leung et al. (2021) [18]	Australia, n = 19	Permanent or rotating night shift workers with abdominal obesity	Randomized crossover trial; 4 weeks; Overnight fasting between 01:00–06:00 h	No differences in postprandial triglyceride and glucose response between intervention and control groups. The overnight fast was well-tolerated by shift workers with an adherence rate of 95%. Lower mean of body weight post intervention compared to post control: −0.9 kg, 95% CI: −1.3 to −0.4.
Manoogian et al. (2022) [19]	USA, n = 137	24 h shifts started at 8 a.m.	Randomized clinical trial; 12-weeks; 10 h TRE.	10 h TRE was feasible with an adherence rate of 73%. Decreased eating window (baseline, mean 14.13 h, 95% CI 13.78–14.47 h; intervention, 11.13 h, 95% CI 10.73–11.54 h, *p* = 3.29 × 10^−17^) Improved quality of life. TRE group significantly decreased VLDL particle size. Participants with elevated cardiometabolic risks at baseline: reductions in glycated hemoglobin A1C and diastolic blood pressure in TRE group.
Teixeira et al. (2023) [111]	Brazil, n = 10	Night workers	Randomized, three-condition, crossover trial; Three different conditions: Night Shift Fasting -two nights of fasting during the night shift; Night Shift Eating—two nights with the consumption of a standardized meal during the night shift (678 ± 42 kcal consumed at ~02:00 h); and Nighttime Sleep—two nights of sleep.	Fasting during the night shift led to a higher intake of energy (~1000 kcal) and macronutrients both in the early morning after work and throughout the next day. Hunger levels were lower after fasting condition compared to the night sleep condition (*p* = 0.012). Insulin and HOMA-IR were also lower in the morning after fasting condition (*p* < 0.001).

## Data Availability

Not applicable.
